# Function of Cryopreserved Cat Ovarian Tissue after Autotransplantation

**DOI:** 10.3390/ani9121065

**Published:** 2019-12-02

**Authors:** Janice M. V. Vilela, Ellen C. R. Leonel, Liudimila P. Gonçalves, Raísa E. G. Paiva, Rodrigo S. Amaral, Christiani A. Amorim, Carolina M. Lucci

**Affiliations:** 1 Departamento de Ciências Fisiológicas, Instituto de Ciências Biológicas, Campus Universitário Darcy Ribeiro, Universidade de Brasília, Brasília 70910-900, Distrito Federal, Brazil; janice.vilela@gmail.com (J.M.V.V.); ellenleonel@yahoo.com.br (E.C.R.L.); liudimila@gmail.com (L.P.G.); raisaegp@gmail.com (R.E.G.P.); 2 Institut de Recherche Expérimentale et Clinique, Pôle de Recherche en Gynécologie, Université Catholique de Louvain, Avenue Mounier, 52 bte B1.52.02, 1200 Brussels, Belgium; christiani.amorim@uclouvain.be; 3 Departamento de Biologia, Instituto de Biociências, Letras e Ciências Exatas (IBILCE), Universidade Estadual Paulista (UNESP), Rua Cristóvão Colombo, 2265, São José do Rio Preto 15054-000, São Paulo, Brazil; 4 Instituto Federal de Educação, Ciência e Tecnologia do Amazonas, Campus Manaus Zona Leste—IFAM/CMZL—Avenida Cosme Ferreira, 8045, Manaus 69083-000, Amazonas, Brazil; rsamaral@gmail.com

**Keywords:** ovary, cryopreservation, transplant, antral follicle, felids

## Abstract

**Simple Summary:**

Assisted reproduction techniques are potentially important tools for the creation of gene banks largely focused on preserving female germ cells and tissues, cryopreservation being one of the most important. Since there is not yet a protocol established for the preservation of cat ovarian tissue, we decided to assess our cryopreservation protocol with autotransplantation of the ovary. Our study showed that even though follicular survival was low, follicles were able to survive up to 28 days of transplantation and develop up to the antral stage, which helps elucidate the path for preservation of felid ovaries. Once this technique is improved, it may contribute to the preservation of wild feline species.

**Abstract:**

The aim of this study was to assess a slow-freezing protocol of cat ovarian tissue cryopreservation using autotransplantation. Four adult queens were ovariohysterectomized and the ovaries were fragmented and cryopreserved. After one week, the grafts were thawed and autografted to the subcutaneous tissue of the dorsal neck of each queen, then randomly removed after 7, 14, 28, 49, and 63 days after transplantation. Percentages of morphologically normal primordial and growing follicles (MNFs) were 88% and 97%, respectively, in fresh tissue samples (fresh controls), and 74% and 100%, respectively, immediately after thawing (cryo D0). No MNFs were found after 49 days of transplantation. In both fresh control and cryo D0 fragments, granulosa cells were frequently in proliferation. Two morphologically normal antral follicles were detected in one queen on Day 28 post-transplantation. Connective tissue fibers increased, suggesting replacement of active ovarian cortex by fibrous tissue. Tissue vascularization was observed at 7 days after grafting, and wide blood vessels were clearly visible on Days 49 and 63. In conclusion, although follicular survival was low after cryopreservation and grafting of cat ovarian tissue, follicles were able to develop up to the antral stage, which is an encouraging outcome.

## 1. Introduction

The leading aim of ovarian tissue cryopreservation is to maintain the viability of oocytes from preantral follicles, a promising approach that has been studied in many species [1,2,3]. In addition to showing normal morphology, cryopreserved tissue samples must be able to resume functionality, which has been successfully assessed by auto or xenotransplantation [4,5]. To date, restoration of ovarian function has been demonstrated in mice [6], sheep [7,8], cows [9], goats [10], and rabbits [11], and more than 130 live births have been reported in humans [12] after transplantation of cryopreserved tissue.

Although some attempts have been made to cryopreserve cat ovarian tissue [13,14,15,16,17,18,19,20,21], the protocols have not yet been well established. Working towards this goal, we have been studying techniques of cryopreservation and restoration of cat ovarian tissue function in our laboratory [21,22,23]. In a previous study, we compared slow-freezing protocols using 1.5 M ethylene glycol (EG), 1.5 M dimethyl sulphoxide (DMSO), and the combination of both (0.75 M each) for cryopreserving cat ovarian tissue. After thawing, we observed better ultrastructure preservation of ovarian follicles cryopreserved with DMSO than with EG or their combination [21]. We also autografted fresh ovarian tissue to the dorsal neck of queens and observed antral follicles on Days 28, 49, 63, and even at 233 days post-transplantation. Additionally, estradiol peaks and estrous behavior were associated with the presence of those follicles [23].

Considering the promising results obtained in both procedures, in the present study, we aimed to assess the function of cryopreserved cat ovarian tissue after heterotopic autotransplantation. Although follicular survival was low, two antral follicles were observed 28 days post-transplantation, which is an encouraging outcome.

## 2. Materials and Methods

### 2.1. Animals

This study was conducted in Brasília, Brazil, a tropical region with no significant change in day length throughout the year (making cats continuously polyestrous [24]). Four healthy mixed-breed adult cats between 1.5 and 3 years of age (2.5 to 4 kg) were used. They tested negative (Anigen Rapid FIV/FeLV Test Bioeasy, Alere, Ref. 34282, São Paulo, Brazil) for feline immunodeficiency virus (FIV) and feline leukemia virus (FeLV). Then, they were de-wormed, acclimated to the environment, and clinically observed for one month before starting the experiment. During the experiment, the cats were housed in individual cages (80 × 60 × 45 cm), with water and standard commercial cat food (Sabor & Vida, Guabi Pet Care, Campinas, Brazil) ad libitum. The cages were located in a ventilated room with 11–13 daily hours of natural light. After the experiment was finished, all cats were adopted. All procedures were approved by the Animal Ethics Committee of the Institute of Biological Sciences, University of Brasilia (protocol #76940/2012).

### 2.2. Ovariohysterectomy

The animals were subjected to bilateral ovariohysterectomy at a local veterinary clinic according to our previously described procedure [23]. Before surgery, they were fasted for 12 h and then were administered meperidine (i.m., Dolosal 50 mg/mL, Cristália, Brazil; 5 mg/kg) and acepromazine (i.m., Acepran 1%, Vetnil, São Paulo, Brazil; 0.2 mg/kg) [25,26]. Midazolam hydrochloride (Midazolam 1 mg/mL, Richmond VetPharma, Buenos Aires, Argentina; 0.5 mg/kg) and ketamine chloride (Cetamin 10%, Syntec, São Paulo, Brazil; 3 mg/kg) were used for inducing general anesthesia. Anesthesia was maintained by ventilation with isoflurane in pure oxygen [25,26].

Ovariohysterectomy was performed according to Fossum’s technique [27]. Each animal received a prophylactic dose of an oral antibiotic (enrofloxacin, Baytril 15 mg, Bayer, Rio de Janeiro, Brazil; 5 mg/kg) and an oral anti-inflammatory (ketoprofen, Ketofen, Merial, São Paulo, Brazil; 2 mg/kg). Fat tissue and ligaments were removed from both ovaries and four pieces of the same size (10 × 3 × 3 mm) were cut from each ovary, totalizing eight pieces per animal. One piece was randomly chosen and immediately fixed in 4% paraformaldehyde as control (fresh control—Day 0). The other pieces were taken to the laboratory in M-199 supplemented with 10% fetal bovine serum (FBS) at 10–12 °C within 40 min.

### 2.3. Slow-Freezing and Thawing of Fragments

Ovarian tissue samples were cryopreserved as described by Leonel et al. [21]. Briefly, fragments were placed in pairs into cryotubes containing 1 mL M-199 with 1.5 M DMSO, 10% FBS and 0.4% sucrose. Cryotubes were equilibrated at 10 °C for 10 min and transferred to a programmable freezer (Dominium K, Biocom, Uberaba, Brazil), where they were cooled at −2 °C/min down to −7 °C and maintained at this temperature for 15 min for seeding. After seeding, they were cooled at −0.3 °C/minute to −35 °C before being immersed into liquid nitrogen (−196 °C).

After 7 days, samples were thawed. First, cryotubes were kept at room temperature for 1 min, after which they were dipped into a water bath at 37 °C until the solution was completely thawed. For cryoprotectant removal, each sample was washed three times, for 5 min each time, in M-199 containing 10% FBS and decreasing concentrations of sucrose (0.4%, 0.2%, and 0%) and DMSO. (0.75 M, 0.375 M, and none).

### 2.4. Autografting

Immediately after thawing, one fragment was fixed in 4% paraformaldehyde (cryo D0). The remaining six were washed in 1% iodine and then three times in 0.9% sterile saline solution before grafting [23]. For both placement and removal of the fragments, the animals were sedated with intravenous ketamine (Cetamin 10%, Syntec, São Paulo, Brazil; 5 mg/kg) and xylazine (Calmium 2%, Agener União, São Paulo, Brazil; 0.5 mg/kg).

For placement, six 1 cm incisions were made in the skin of the dorsal neck region, creating a small pouch (~1 cm^3^) in the subcutaneous tissue. One fragment was randomly placed inside each pouch and the incision was closed. Each queen received 5 mg/kg vitamin E (i.m., Monovin E—Bravet, Rio de Janeiro, Brazil) to reduce the ischemic damage that occurs after transplantation [28,29].

The grafts were randomly removed after 7, 14, 28, 49 (one graft per day), and 63 days (the remaining two grafts), and fixed in 4% paraformaldehyde for analyses.

### 2.5. Histological, Immunohistochemical and Histochemical Analyses

Ovarian tissue fragments were dehydrated in ethanol, clarified in xylene, and embedded in Paraplast Plus^®^ (Merck, Ref. P3683, São Paulo, Brazil). Every fourth section (4 μm thickness) was stained with hematoxylin and eosin (HE; Merck, Darmstadt, Germany) and used for follicles counting. If there were any doubts about the classification of a particular follicle/structure seen in these sections, adjacent sections were stained, and consulted to ensure classification. Of the remaining sections, some were selected for Masson’s trichrome staining and immunohistochemistry.

During evaluation of HE-stained sections, typical follicles were classified as primordial (with one layer of flattened granulosa cells surrounding the oocyte) or growing (with one or more layers of cuboidal granulosa cells surrounding the oocyte) [30]. They were also categorized as morphologically normal or degenerated. Morphologically normal follicles (MNFs) showed a uniform distribution of granulosa cells and a spherical oocyte; otherwise, they were considered degenerated. To avoid double-counting typical follicles, only those with a visible oocyte nucleus were considered.

Masson’s trichrome staining, with which collagenous connective tissue is stained in green, was used to detect fibrotic areas in grafts. Terminal deoxynucleotidyl transferase (TdT) dUTP Nick-End Labeling (TUNEL) assay, Ki67 and CD31 markers were used to identify apoptosis, cell proliferation and vascularization, respectively.

TUNEL assay was performed using the In Situ Cell Death Detection Kit, TMR Red (Roche, REF 12 156 792 910, Mannheim, Germany) as described by Vanacker et al. [31]. Human tonsil tissue was used as positive control, and negative control sections were incubated with label solution but without enzyme solution. Slides were counterstained with DAPI and examined under a fluorescence microscope (Leica; Van Hopplynus Instruments, Brussels, Belgium). Red fluorescence was visualized in TUNEL-positive cells by applying excitation and emission wavelengths in the range of 520–560 nm and 570–620 nm, respectively. DAPI reached excitation and emission wavelengths at about 360 nm and 460 nm, respectively, when bound to DNA, emitting blue fluorescence. Classification of follicles was based on the percentage of dead cells according to Martinez-Madrid et al. [32].

Cell proliferation analysis was conducted with mouse anti-human Ki-67 IgG (4 °C, 1:50 dilution; clone MIB-1, REF M7240, Dako, Glostrup, Denmark) and goat anti-mouse secondary antibody (Dako, K4001, Glostrup, Denmark), as previously described by Dolmans et al. [33]. Human proliferative endometrium was used as positive control, while the negative control consisted of the dilution solution without primary antibody. Follicles with at least one Ki67-marked granulosa cell were considered as proliferative.

The protocol described above was also applied for vascularization assessment, with rabbit anti-CD31 (PECAM-1 human clone EP3095, mAb, Epitomics, Ref 2530-1, Burlingame, CA, USA) as the primary antibody and goat anti-rabbit as the secondary antibody (Dako, K4003, Glostrup, Denmark).

### 2.6. Statistical Analyses

Treatment groups were compared to each other by *t*-test and Mann–Whitney (for growing degenerated follicles) analyses using SPSS software version 17.0. A *p* value < 0.05 was considered significant.

## 3. Results

### 3.1. Graft Retrieval

By Day 7, grafts were not yet fully adhered to the subcutaneous tissue and could be easily removed. After 14 days, however, all samples but one were well attached, and after 28 days, all had adhered and were enclosed in a fibrous capsule. From Day 14 onward, recovered fragments were smaller in comparison to the day they were grafted and most were round-shaped. By Day 28, they were palpable under the skin, but harder to visualize within the subcutaneous tissue when the pouch was opened. By the end of the experiment, only 2 out of 24 grafts (8.3%) failed to be recovered. Ultrasonographic examination of the transplantation site did not reveal the two missing fragments, suggesting that they had been reabsorbed.

### 3.2. Microscopic Aspect of the Grafts

**Figure 1:**
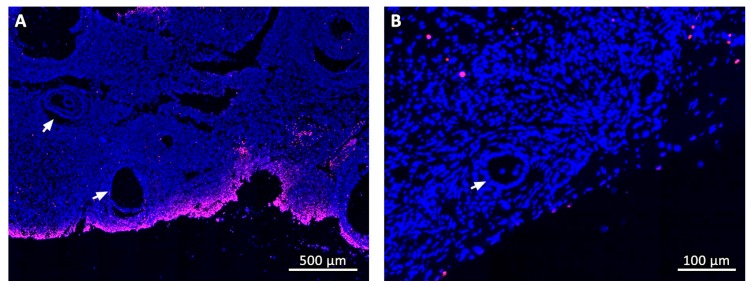
TUNEL assay. General aspect of the tissue in a cryo D0 fragment (**A**) and on day 14 after grafting (**B**). Dead cells are stained in red fluorescence. White arrows point to ovarian follicles.

**Figure 2:**
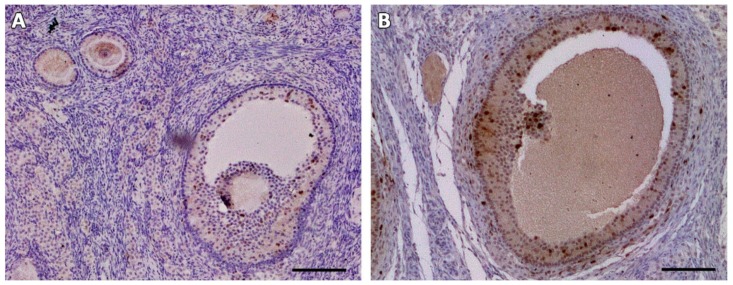
Ki67 staining (in brown) in follicles in the fresh (**A**) and cryo D0 (**B**) groups. Bars: 100 µm.

Numbers and percentages of MNFs and degenerated follicles were not significantly different between fresh control and cryopreserved tissue at Day 0 (cryo D0) (Table 1), but the total number of follicles in both groups was drastically reduced after grafting. Moreover, most follicles had degenerated and very few growing follicles were detected after transplantation. MNF percentages at each time-point post-grafting were lower than fresh control and cryo D0 (*p* < 0.05). TUNEL assays showed that follicles found immediately after thawing (cryo D0) and on Days 7, 14, and 28 post-grafting were not dead (Figure 1). In fresh control and cryo D0 fragments, most follicles (primordial and growing) exhibited Ki67-positive granulosa cells (Figure 2).

**Figure 3:**
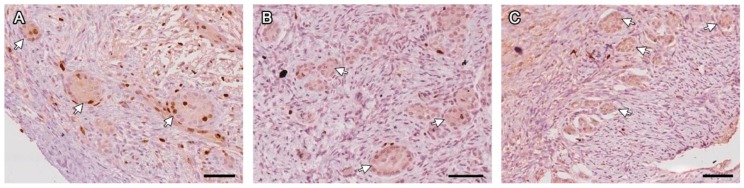
FLSs with juxtaposed granulosa cells and no oocyte (white arrows) labeled with Ki67 on days 7 (**A**), 14 (**B**) and 28 (**C**) post-grafting. Bars: 200 µm.

A common finding in all treatment groups (Table 2) were follicle-like structures showing juxtaposed granulosa cells without an oocyte, which were considered a type of degeneration after confirming there was no oocyte in any adjacent section. Interestingly, besides the absence of an oocyte, it was confirmed by Ki67 that these granulosa cells were proliferating (Figure 3). Other types of degeneration included oocyte pyknotic nuclei, ooplasmic vacuoles, retracted or detached oocytes, and follicles detached from the stroma.

**Figure 4:**
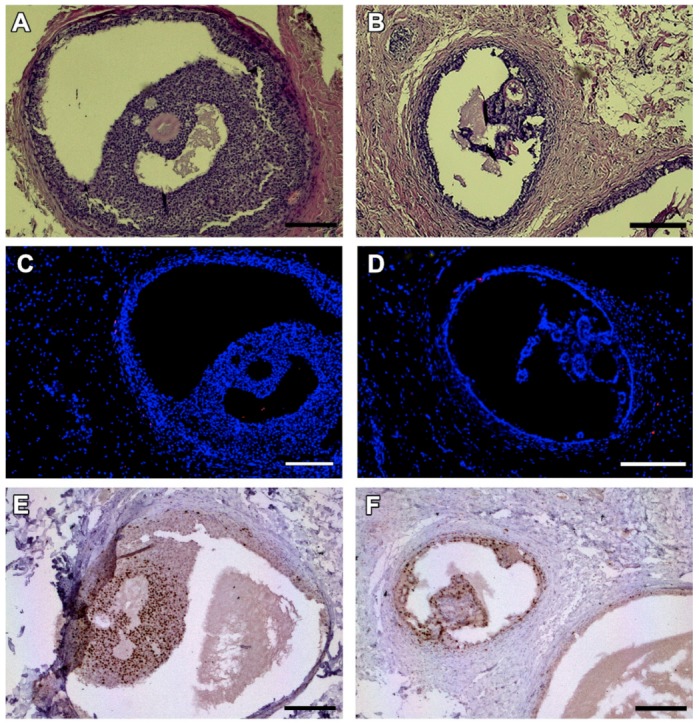
Antral follicles found in one animal on day 28 after grafting. HE-staining (**A**,**B**). TUNEL assay shows no dead granulosa cells (no red fluorescence) (**C**,**D**). Ki67 staining reveals proliferating granulosa cells stained in brown (**E**,**F**). Bars: 200 µm.

In one animal, two antral follicles were found on Day 28 after grafting (Figure 4A,B). Both were viable, as evidenced by TUNEL assay (Figure 4C,D) and Ki67 staining (Figure 4E,F). No MNFs were detected in any animals after 14 days.

**Figure 5:**
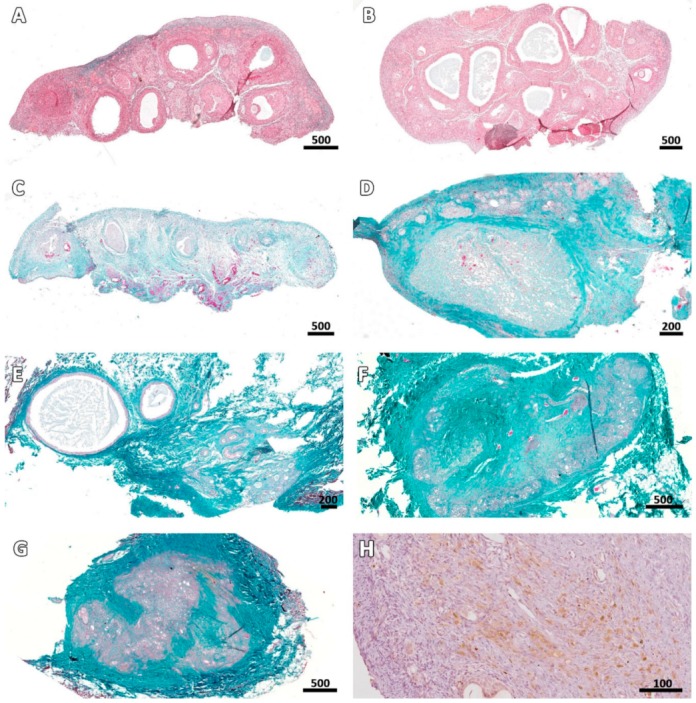
Ovarian stroma at different stages post-transplantation. Masson’s trichrome staining shows an increase in the extracellular matrix (collagen fibers stained green) of connective tissue in fresh fragments (**A**) and cryopreserved fragments immediately after thawing (**B**), on Days 7 (**C**), 14 (**D**), 28 (**E**), 49 (**F**), and 63 (**G**) after grafting. Ki67 staining illustrates stromal cell proliferation on Day 28 (**H**). Bars: scale in µm.

Masson’s trichrome revealed that the deposition pattern of collagen fibers varied between ovarian samples before (fresh control and cryo D0, Figure 5A,B) and after transplantation. From Day 7 onwards, most stromal tissue areas visibly contained an abundance of connective fibers and their cellularity was considerably reduced, especially in central areas of the grafts (Figure 5C–G). Nevertheless, Ki67 staining showed that stromal cells were still proliferating in all fragments (Figure 5H). TUNEL assays found no dead cells in fresh controls, while on cryo D0 samples, the periphery tissue was stained red (Figure 1), suggesting that this region suffered some damage during slow-freezing and/or thawing.

**Figure 6:**
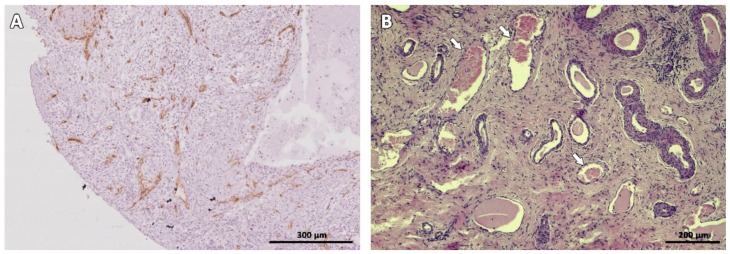
Vascularization of ovarian tissue marked with CD-31 immunostaining (brown) on Day 7 (**A**) and HE staining on Day 63 after transplantation (**B**). White arrows indicate blood vessels.

Tissue vascularization was observed 7 days post-transplantation, as demonstrated by CD31 immunoreactivity (Figure 6A), and large blood vessels were clearly visible on Days 49 and 63 post-grafting (Figure 6B).

## 4. Discussion

In the present study, we used autotransplantation to evaluate the survival and development of preantral follicles and tissue quality after cat ovarian tissue cryopreservation. The results showed a drastic reduction in follicle population and increased fibrosis in ovarian tissue. Even so, remaining follicles were able to resume their development up to antral stage, which is an encouraging outcome.

After freezing and thawing, numbers and percentages of MNFs detected were not significantly different from those found in fresh control samples, which is consistent with our previous findings [21]. Our results are similar to the ones reported on vitrification of cat ovarian tissue using DMSO supplemented with sucrose [18]. Using the same cryoprotectants, Tanpradit et al. [16] reported better results in slow freezing than vitrification of cat ovarian tissue for follicular viability, histology, and apoptosis assessment.

While normal morphology is an important criterion, it does not translate to viability, and thus it is necessary to use a method that allows follicle activity reestablishment to actually evaluate the cryopreservation protocol. Indeed, we can clearly see this difference when we compare our present findings with our previous study on autografting of fresh ovarian tissue [23]. Despite the high proportion of MNFs in cryopreserved tissue at Day 0 (cryo D0), post-transplantation follicle survival rates were lower than those reported by Leonel et al. [23], who found more than 90% of MNFs on Day 28 after grafting fresh ovarian tissue, and a mean of 181 follicles per fragment on all grafting days. Since the cryopreservation protocol and the transplantation technique applied were the same as previously described [21,23], this suggests that cryopreserved follicles have lower survivability after transplantation than fresh follicles.

The most significant type of degeneration observed was the presence of follicle-like structures (FLSs) containing juxtaposed granulosa cells without an oocyte. This degeneration was also encountered by Leonel et al. [23] from Day 7 after transplantation of fresh cat ovarian tissue, which indicates that it is probably an effect of the grafting procedure. In the present study, FLSs were the only structures observed from Day 14 onwards, which shows that they can survive for several days without an oocyte. Oocyte degeneration was reported as the most frequent sign of atresia in preantral follicles [34,35,36,37], while granulosa cells continued to survive and proliferate [36,37], demonstrating that cryopreserved oocytes are much more sensitive to adverse conditions than granulosa cells.

Although most follicles did not survive, one cat presented two antral follicles that were alive and showed proliferating granulosa cells 28 days after transplantation (Figure 4). The time required for antral follicles development in cats is unknown, but on in vitro culture, 50% of isolated secondary follicles showed an antral cavity after 14 days [38]. Considering that growing follicles are more susceptible to hypoxic conditions before tissue reperfusion [39], it is likely that the antral follicles detected 28 days after grafting derived from primordial follicles developing after transplantation.

After transplantation of cryopreserved ovarian tissue, a visible change in connective fibers and cellularity patterns was observed, especially in the central areas of the grafts. Previous studies have reported the negative impact of freezing on ovarian stromal cells [40,41], and in cats; Bosch et al. [13] also found a reduction on stromal cellularity 67 days after xenotransplantation of ovarian tissue. Indeed, production of TGF-β1 by fibroblasts in response to hypoxia may be an important factor for collagen and other extracellular matrix synthesis, leading to fibrosis [42,43]. The size of the fragments might also have affected the extension of fibrosis in the transplanted tissue [44].

Even with low follicle recovery rates, increased vascularization was observed in the grafts on all evaluated days, and large blood vessels could be seen on Days 49 and 63 post-transplantation. It is known that the most extensive follicle loss occurs during the period of ischemia that develops before tissue reperfusion and revascularization. This period varies among species, from 48 h in rats [45], to 5 days in humans [46,47], and 7 days in sheep [48]. The time needed for cat ovarian tissue to become vascularized after transplantation is not known, and studies are required to elucidate this process.

Development of ovarian follicles after transplantation of cryopreserved ovarian tissue has been reported in cows [9], goats [10] and human [49]; embryo development in rabbits [11] and sheep [8]. Moreover, live births were reported in rhesus monkeys [50], humans [51,52], and mice [53]. Cryopreservation of felid tissue, in turn, appears to show particularly inferior performance compared to other species. While xenotransplantation of fresh ovarian tissue to immunodeficient mice has shown follicle survival rates of up to 54%, antral follicle development and germinal vesicle breakdown [54,55], xenografting of cryopreserved ovarian tissue showed a 10% survival rate. Still, antral follicles were obtained 67 days after transplantation [13]. In wild felids, low rates of follicle activation and development were observed after xenografting of lioness cryopreserved ovarian tissue to immunodeficient mice [56]. Thus, the present study corroborates the literature on cryopreservation of cat ovarian tissue, helping to elucidate the path towards developing specific protocols for cat ovarian tissue cryopreservation and transplantation.

## 5. Conclusions

Despite tissue revascularization observed from Day 7 onwards, there was a low follicle recovery rate, probably due to the combination of injuries caused by cryopreservation with the damage caused by ischemia-reperfusion period in the early days of the tissue transplant. Nevertheless, two antral follicles were recovered after transplantation of cryopreserved ovarian tissue. Alternative grafting sites and strategies to reduce the period of ischemia after transplantation, as well as improvements to the cryopreservation method, are being investigated in our group to improve and promote follicular survival and development of cryopreserved cat ovarian tissue. Once these techniques are improved, they may contribute to the preservation of wild feline species, since the domestic cat is an excellent experimental model for developing techniques to be used in wild cats.

## Figures and Tables

**Table 1:** Number and percentage of primordial and growing follicles found on fresh tissue (fresh controls), cryopreserved tissue immediately after thawing (cryo D0) and 7, 14, 28, 49, and 63 days after grafting.

Type	Treatment	Normal		Degenerated		Total
		Total	%	Total	%	Follicles
**Primordial**	Fresh controls	491 ^a^	87.8 ^a^	68 ^a^	12.2 ^a^	559 ^a^
	Cryo D0	471 ^a^	73.7 ^a^	168 ^a^	26.3 ^a^	639 ^a^
	7 days	31 ^b^	31.3 ^b^	68 ^a^	68.7 ^b^	99 ^b^
	14 days	2 ^b^	3.3 ^b^	59 ^a^	96.7 ^b^	61 ^b^
	28 days	11 ^b^	26.8 ^b^	30 ^a^	73.2 ^b^	41 ^b^
	49 days	0 ^b^	0.0 ^b^	10 ^b^	100.0 ^b^	10 ^b^
	63 days	0 ^b^	0.0 ^b^	9 ^b^	100.0 ^b^	9 ^b^
**Growing**	Fresh controls	29 ^ab^	96.7	1	3.3	30 ^ab^
	Cryo D0	36 ^b^	100.0	0	0.0	36 ^b^
	7 days	2 ^ac^	40.0	3	60.0	5 ^ac^
	14 days	0 ^c^		0		0 ^c^
	28 days	2 ^ac^	100.0	0	0.0	2 ^ac^
	49 days	0 ^c^		0		0 ^c^
	63 days	0 ^c^		0		0 ^c^

^a,b,c^ Values with different superscripts in the same column are statistically different (*p* < 0.05), analyzed separately for Primordial and Growing follicles.

**Table 2:** Percentage of FLSs with juxtaposed granulosa cells and no oocyte relative to degenerated follicles found (% FLSs/DFs) and total follicles counted (% FLSs/TFs).

Treatment	% FLS/DFs	% FLS/TFs
Fresh controls	1.4 (1/69)	0.2 (1/589)
Cryo D0	8.9 (15/168)	2.2 (15/675)
7 days	63.4 (45/71)	43.3 (45/104)
14 days	100.0 (59/59)	96.7 (59/61)
28 days	100.0 (30/30)	69.8 (30/43)
49 days	100.0 (10/10)	100.0 (10/10)
63 days	100.0 (9/9)	100.0 (9/9)

No statistical analysis was performed. FLSs: Follicle-like structures; DFs: Degenerated follicles; TFs: Total follicles.
